# Differential Toxicity of Antibodies to the Prion Protein

**DOI:** 10.1371/journal.ppat.1005401

**Published:** 2016-01-28

**Authors:** Regina R. Reimann, Tiziana Sonati, Simone Hornemann, Uli S. Herrmann, Michael Arand, Simon Hawke, Adriano Aguzzi

**Affiliations:** 1 Institute of Neuropathology, University of Zurich, Zurich, Switzerland; 2 Institute of Pharmacology and Toxicology, University of Zurich, Zurich, Switzerland; 3 Vascular Immunology Laboratory, Department of Pathology, University of Sydney, Camperdown, Australia; University of Edinburgh, UNITED KINGDOM

## Abstract

Antibodies against the prion protein PrP^C^ can antagonize prion replication and neuroinvasion, and therefore hold promise as possible therapeutics against prion diseases. However, the safety profile of such antibodies is controversial. It was originally reported that the monoclonal antibody D13 exhibits strong target-related toxicity, yet a subsequent study contradicted these findings. We have reported that several antibodies against certain epitopes of PrP^C^, including antibody POM1, are profoundly neurotoxic, yet antibody ICSM18, with an epitope that overlaps with POM1, was reported to be innocuous when injected into mouse brains. In order to clarify this confusing situation, we assessed the neurotoxicity of antibodies D13 and ICSM18 with dose-escalation studies using diffusion-weighted magnetic resonance imaging and various histological techniques. We report that both D13 and ICSM18 induce rapid, dose-dependent, on-target neurotoxicity. We conclude that antibodies directed to this region may not be suitable as therapeutics. No such toxicity was found when antibodies against the flexible tail of PrP^C^ were administered. Any attempt at immunotherapy or immunoprophylaxis of prion diseases should account for these potential untoward effects.

## Author Summary

The human prion disease, Creutzfeldt-Jakob disease (CJD), is a progressive neurodegenerative syndrome. Although far less prevalent, CJD shows many molecular and clinical similarities to Alzheimer's disease, such as the buildup of protein aggregates in the brain and the absence of effective treatments. Many attempts at immunotherapy for Alzheimer’s disease are being reported in specialized journals and in the lay press, and have been linked to strong hopes for a cure. The same therapeutic strategy appears plausible for Creutzfeldt-Jakob disease, and indeed, there are some encouraging preclinical studies. However, there have also been reports that antibodies against the prion protein (PrP^C^) can also wreak damage on the brain. We have gathered evidence that various antiprion antibodies vary not only in their efficacy but also in their potential to induce serious untoward effects. In a dose-escalation study, we report that all antibodies against a set of epitopes in the globular domain of the prion protein display acute neurotoxicity. These issues need to be carefully assessed before considering any clinical studies involving human subjects.

## Introduction

Active and passive immunotherapy that foster the clearance of pathological aggregates represent potential therapeutic strategies against diseases caused by the inappropriate aggregation of proteins [1]. While considerable effort has been devoted to the immunotherapy of Alzheimer's disease with antibodies against the Aβ protein [2, 3], transmissible spongiform encephalopathies (TSE) represent equally plausible candidates for this approach. TSEs are caused by self-propagating aggregates of PrP^Sc^, a conformer of the cellular prion protein PrP^C^ encoded by the *Prnp* gene. Active immunotherapeutic strategies in preclinical disease models have rarely yielded significant improvements in survival time after prion inoculation [4, 5]. In addition, it has proven difficult to induce high-affinity immune responses to PrP^C^ in wild-type mice even in the presence of a variety of adjuvants [6].

Passive immunotherapeutic strategies may be more likely to succeed. A first proof of concept for immunotherapies in prion disease was established with mice genetically engineered to express the heavy chain of an anti-PrP^C^ antibody. These mice were found to be protected against peripheral prion infection [7]. Later, it was observed that passive intraperitoneal immunization with antiprion antibodies ICSM18 and ICSM35 blocked peripheral infection with Rocky Mountain Laboratory strain mouse-adapted scrapie prions (RML), although no beneficial effect was seen upon intracerebral inoculation [8]. With intravenous delivery of the antibodies 31C6, 110 and 44B1, a trend towards longer survival could be detected after intracerebral inoculation of the Chandler and Obihiro prion strains [9]. Additionally, osmotic minipumps were used to deliver antibody 31C6 intraventricularly, and this intervention led to a significant prolongation of survival in mice inoculated with prions intracerebrally [10]. Table 1 summarizes the features and outcomes of preclinical active and passive immunization attempts that have been published thus far.

**Table 1:** Preclinical active and passive immunization against prion disease.

**Study design**	**Antibody**	**Outcome**	**Reference**
Transgenic expression of antiprion antibodies	6H4	Protection against i.p. RML prion inoculation	[7]
Active immunization with PrP peptides	PrP_131–150_	Immunogenic response and reduction of PrP^Sc^ in tumor transplants	[11]
	PrP_211–250_		
Active vaccination with recombinant mouse prion protein	Full length rPrP	Prolonged latency and clinical course after i.p. 139A prion inoculation	[5, 12]
Passive immunization with antiprion holoantibodies	ICSM18 ICSM35	Protection against i.p. RML prion inoculation	[8]
Active immunization with PrP peptides	PrP_105–128_	Increased survival in a hamster model of TSE	[4]
	PrP_119–146_		
	PrP_142–179_		
Passive immunization with holoantibodies and F(ab)_1_ fragments delivered by osmotic minipumps	4H11	Severe untoward effects with neuronal cell loss, astrogliosis and microglia activation	[13]
Passive immunization with holoantibodies delivered by osmotic minipumps	110	Partial prolongation of survival after intracerebral inoculation with the Chandler and Obihiro prion strains	[10]
	31C6		
	44B1		
Passive immunization with holoantibodies injected into the tail vein	31C6	Minimal prolongation of survival time	[9]

On the other hand, chronic intracerebral administration of the antiprion antibody 4H11 resulted in severe side effects, including nerve cell loss, gliosis, and microglial activation [13]. Similar toxic side effects were detected by us and others after stereotaxic injection of various anti-PrP^C^ antibodies, apparently strictly dependent on the particular PrP^C^ epitope targeted by the respective antibody [14, 15]. Whilst all of the above findings have raised concerns about the safety of anti-PrP^C^ immunotherapies, Klöhn *et al*. [16] reported that they did not reproduce the neurotoxicity described for antibody D13. Furthermore, they reported no acute toxicity in vivo for their own antibodies ICSM18 and ICSM35. The study by Klöhn *et al*. is surprising, not only because it contradicted earlier reports of D13 toxicity, including our findings of lesions upon injection of D13 into PrP^C^-overexpressing *tg*a*20* mice [17], but also because of our previously published results that 7/12 antibodies to the globular domain of PrP^C^ are acutely neurotoxic [15]. Interestingly, crystallographic studies revealed that ICSM18 and the neurotoxic antibody POM1 share a conspicuous overlap in their respective epitopes. In particular, both antibodies have close intermolecular contacts (<4Å) to the amino acid side chains Ser143, Asp144, Tyr145 and Lys204 of human PrP [18, 19]. The first three amino acids correspond to the murine residues Asn143, Asp144 and Trp145, which are part of the murine PrP binding interface of ICSM18 [20], confirming epitope similarities between the two species. If residues with an interaction distance shorter than 5 Å are included, the shared interface between the two antibodies is even more impressive and encompasses 9 amino acids (S1 Fig).

The apparent discrepancy in the toxicity of POM1 and ICSM18 is of great theoretical and practical interest. Toxic anti-PrP^C^ antibodies induce damage by triggering pathways similar to those detected in *bona fide* prion infections, including activation of calpains and the PERK pathway as well as the production of reactive oxygen species [21]. Mechanistically, the flexible tail at the amino-terminus of the prion protein mediates the toxicity of antiprion antibodies by binding to the globular domain of PrP [15]. Therefore, aside from the obvious issues of safety for human clinical trials, understanding why two antibodies directed against extremely similar epitopes might display such divergent on-target toxicity may advance our understanding of prion pathogenesis. In order to address the above questions, we set out to replicate the experiments described by Klöhn *et al*. In addition, as behooves any systematic toxicological study, we expanded our experiments to include the dose-response analyses that had not been performed in previous studies.

## Results

### Antiprion antibody D13 induces acute neurotoxicity in vivo

**Fig 1:**
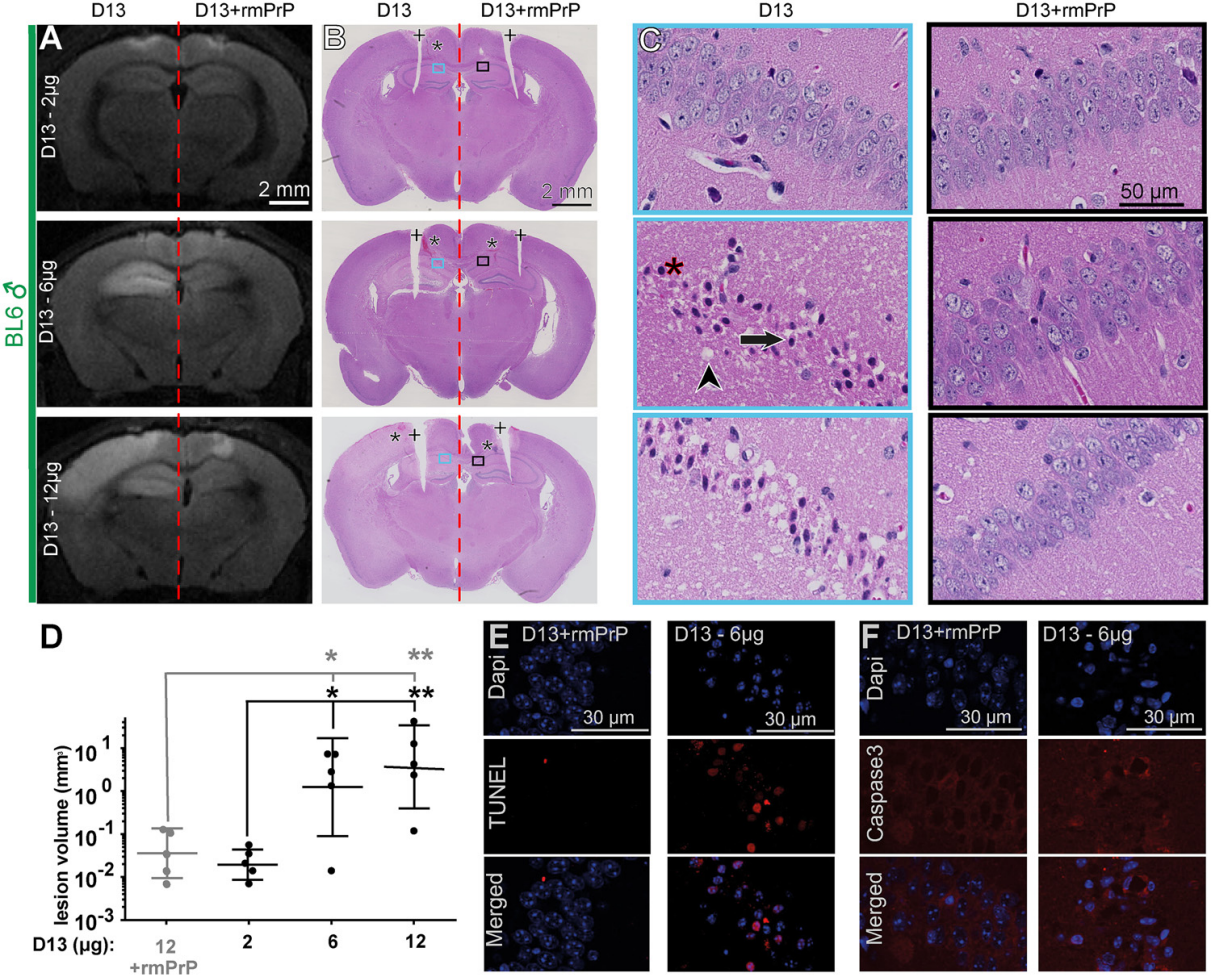
Dose-escalation study of D13. (**A**) Magnetic resonance DWI images at 48h after injection of antibody D13 (2, 6 and 12 μg; respectively) into the left hippocampus. For control, D13 was preincubated with a three-fold molar excess of recombinant mouse PrP (residues 90–230; D13+rmPrP) and injected into the right hippocampus. Whereas both D13 and D13+rmPrP induced minor traumatic lesions in the neighborhood of the injection site, only D13 induced extensive hyperintensity throughout the hippocampus. (**B**) Haematoxylin and eosin (HE) stained sections from the mice shown in panel A. Asterisks: needle track (only visible on select sections). Rectangles denote regions magnified in panel C. The bilateral cortical incisions (crosses) were introduced post-mortem as landmarks in order to properly orient the paraffin blocks for histology. (**C**) Higher magnification of the Cornu ammonis, sector 1 (CA1). Left panels: D13 injections (2, 6 and 12 μg). Right panels: D13+rmPrP. D13-exposed tissue displayed vacuoles indicative of edema (arrowhead). Numerous neurons showed condensed chromatin, hypereosinophilic cytoplasm (asterisks), and nuclear disintegration (arrow). Injection of 12 μg induced neuropil coarsening indicative of severe lesions. (**D**) DWI-based volumetric quantification of lesions depicted on a log_10_ scale after 2, 6 and 12 μg of D13. Statistical analysis revealed significant lesion induction at 6 and 12 μg of D13 in contrast to injection at 2 μg of D13 and D13 (12 μg) preincubated with rmPrP (grey). *N = 5*, mean±sd of log_10_ values_,_ one-Way Anova with Dunnett’s post-hoc test, **P<0.01, *P<0.05. (**E**) TUNEL-stained paraffin sections of the CA1 region of the mouse shown in panels A-C (injection of 6 μg D13). Quantitation showed 41±19% TUNEL^+^ cells (*n *= 30 fields at 20x) after exposure to D13 and ca. 0.5% TUNEL^+^ cells after exposure to D13+recPrP. Blue: nuclear counterstaining with 4’,6-diamidino-2-phenylindole (Dapi). (**F**) Confocal laser scanning microscopy of paraffin sections immunostained for activated caspase-3 revealed little to no caspase activity after injection of 6 μg D13.

We stereotaxically injected antibody D13 (2 μg in 2 μl PBS) into the left Cornu ammonis region-1 (CA1) of the hippocampus of male 3–4 month old C57BL/6 (designated BL6) mice. The coordinates were identical to those used in previous studies with this antibody [14, 16]. For control, D13 antibody pre-incubated (1h, room temperature) with a three-fold molar excess of a recombinant murine PrP fragment encompassing residues 90–231 (rmPrP) was injected into the right (contralateral) hippocampus. Histological examination at 48h post injection (p.i.) and diffusion weighted magnetic-resonance imaging (DWI) 24h p.i. failed to reveal any lesion at either the injection site, other than mild traumatic damage and acute extravasation limited to the immediate vicinity of the stereotaxic needle track (Fig 1A–1C, upper row).

We then reasoned that despite their monoclonal origin, the biological activity of antibodies can vary between batches. Furthermore, even the assessment of protein concentration can vary between labs and can depend on the specific methodology utilized. As the dose dependence of antiprion antibodies (e.g. POM1, D13) has already been demonstrated ex vivo [15]; therefore, we examined the toxicity of D13 over a range of antibody concentrations. Indeed, when 6 μg or 12 μg of D13 were injected, a conspicuous hyperintense lesion became apparent at 48h p.i. by DWI in the hippocampus and/or cortex (Fig 1A, middle and lower row; S1 Table). Again, the contralateral hippocampi were injected with D13 antibody preincubated with its cognate antigen using the procedure described above did not display any DWI signal alteration. Histologically, D13 (48h p.i.) caused conspicuous edema and widespread acute neuronal damage affecting widespread cortical and/or hippocampal areas (Fig 1B and 1C, middle and lower row). Affected neurons displayed condensed hyperchromatic nuclei and hypereosinophilic cytoplasm (Fig 1C, middle and lower). Some neurons showed prominent nuclear fragmentation. We then quantified the hyperintense signal by volumetry (S1 Table): statistical analysis revealed significant lesion induction at 6 μg and 12 μg D13 compared with D13 injection at 2 μg and injection of D13 (12 μg) preincubated with rmPrP (Fig 1D). In order to estimate the upper limit of the D13 intracerebrally injected safe dose, we performed a benchmark dose analysis which yielded a dose of 3.7–5.4 μg (S2A Fig) [22].

It was originally reported [14] that D13 injection results in apoptosis, as visualized by positive labelling for terminal deoxynucleotidyl transferase dUTP nick end labelling (TUNEL)[16]. We found TUNEL-positive cells in lesions of mice injected with 6 μg, but not in the control injections with antigen-preincubated D13 (Fig 1E). In contrast, activated caspase-3 (aC3) immunohistochemistry labelled only a few cells (Fig 1F). This result is in line with the previous report that the neurotoxicity of anti-PrP^C^ antibodies does not lead to caspase activation and cannot be suppressed by caspase inhibition [23].

### ICSM18 triggers mouse hippocampal neurotoxicity

Next, we addressed the possible toxicity of antibody ICSM18 after intracerebral injection. Again we performed stereotaxic inoculations into the left CA1 region of the hippocampus. In order to exclude gender and strain-dependent confounders, we performed this experiment in female C57BL/10 (henceforth designated BL10) mice as in Klöhn *et al* [16]. For control, we administered IgG1 isotype control (BRIC222, 6 μg) into the contralateral stereotaxic position, in order to replicate all details of the Klöhn study [16]. Because only limited amounts of ICSM18 were available, we were unable to perform control experiments with antigen-blocked ICSM18 antibody.

**Fig 2:**
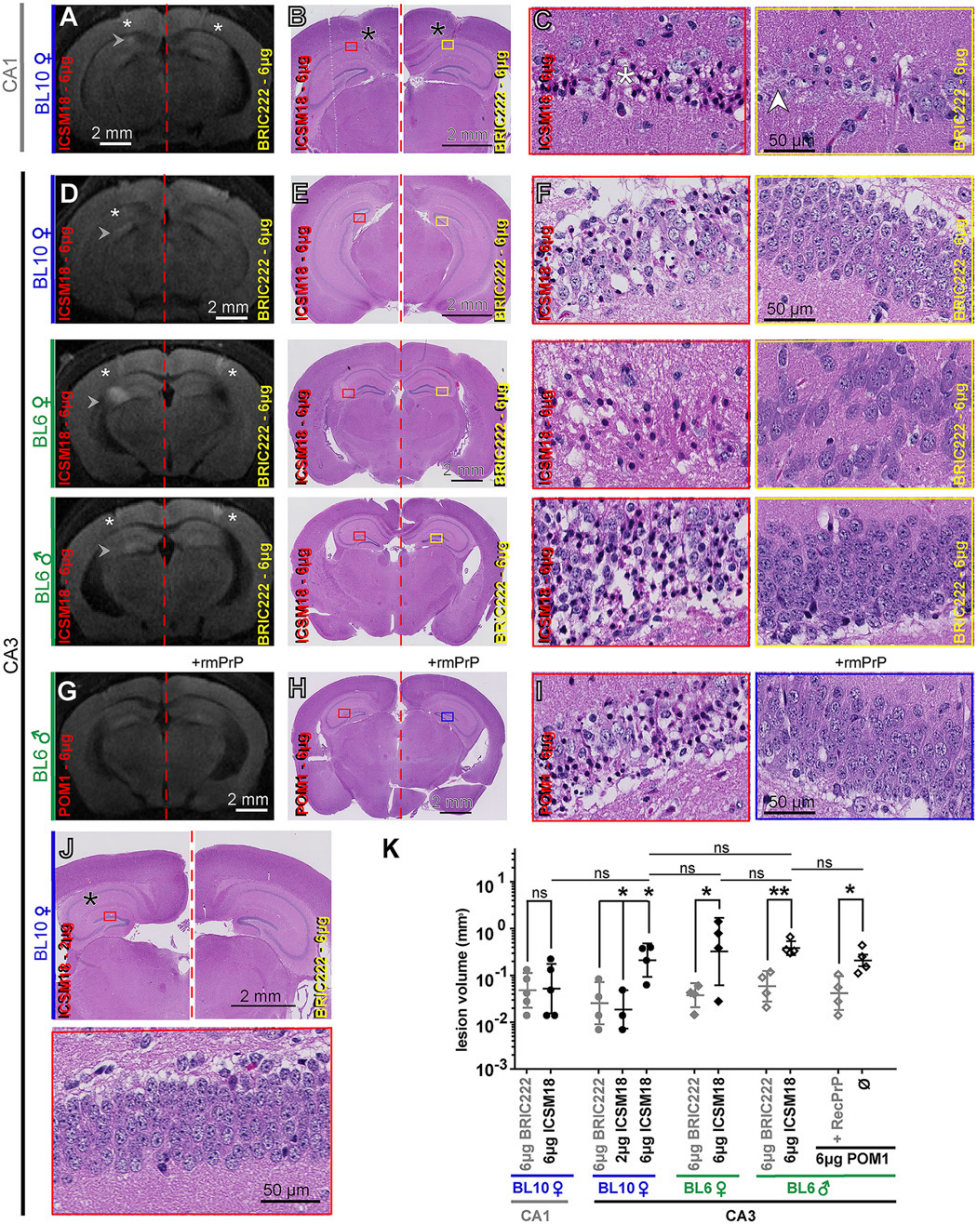
ICSM18 trigger mouse hippocampal neurotoxicity. (**A**) DWI showing a hyperintense lesion (arrowhead) at 24h after injection of ICSM18 (6 μg) into the left CA1 region of a BL10 female mouse. No signal alteration was found on the contralateral side injected with IgG_1_ isotype control. Arrowheads point to hyperintense lesions. Asterisks: needle tracks. (**B**) HE-stained histological sections of the brain shown in panel A (48h p.i.). Asterisks: location of needle track. Rectangles: regions magnified in panel C. (**C**) High-magnification images of the regions identified by the red and yellow rectangles, respectively, on panel B. Neurons with condensed nuclei and hypereosinophilic cytoplasm were found (star) in the area corresponding to the DWI hyperintensity (left panel). In contrast, the mechanical lesions induced by the needle track were characterized by cellular debris (right panel, white arrowhead). (**D**) Representative DWI images 24h after stereotaxic injection of 6 μg ICSM18 versus BRIC222 into the CA3 region of BL6 females, BL10 males, or BL10 females (as indicated). Arrowheads point to hyperintense lesions. Asterisks: needle tracks (only visible on select planes). (**E**) HE-stained histological sections of the brains depicted in panel D. Mice were sacrificed at 48h p.i. Rectangles: regions magnified in Panel F. (**F**) High-magnification images of the regions identified on panel E. Numerous dying neurons are seen in the dentate gyrus after exposure to 6 μg ICSM18 (red rectangle), but not to 6 μg BRIC222 (yellow rectangle). Occasional "dark neurons" were found in BRIC222-treated samples at frequencies similar to those of untreated mice, and were interpreted as fixation artifacts. (**G**) 24h after CA3 administration of 6 μg POM1, the findings were similar to those after ICSM18 administration. For control, we blocked POM1 by pre-incubation with a three-fold molar excess of the antigen rmPrP. (**H**) HE micrograph from the mouse depicted in panel G (48h p.i.). Rectangles: regions magnified in Panel I. (**I**) POM1 related tissue damage was morphologically similar to the ICSM18-induced neurotoxicity shown in panel F. (**J**) Representative HE images at high and low resolution 48h p.i. of 2 μg ICSM18 versus 2 μg BRIC222. No lesions were found. (**K**) Significant lesion induction was found after injection of 6 μg ICSM18 into the CA3 region of female BL10 mice, but not after injection into the CA1 region and not after injection of 2 μg. Lesion volumes were slightly larger in BL6 mice of either gender (two-tailed Student’s *t*-test). Stereotaxic injection of POM1 (same dose as ICSM18) induced damage similarly to the injection of ICSM18 in BL6 mice. Log_10_ scale; *n = 5* for CA1 region injections and *n = 4* for CA3 injections; mean ±SD of log_10_ values; Multi column comparison (sample three to five) with one-way Anova with Tukey’s post-hoc test, comparing of two samples with two-tailed Student’s *t*-test, **P<0.01, *P<0.05, ns: not significant.

DWI visualized a small lesion 24h p.i. in 1/5 mice injected with 6 μg of ICSM18, whereas no lesions were seen in the control group (Fig 2A, S1 Table). Serial sections (48h p.i.) stained with haematoxylin-eosin (HE) revealed a lesion histologically similar to those observed after D13 injection and eminently distinguishable from the traumatic needle track damage by the presence of widespread condensed nuclei (Fig 2B and 2C). This finding raised concern that ICSM18 might be neurotoxic, yet statistical analysis failed to reveal significant differences between ICSM18 injections and contralateral isotype control injections in BL10 mice (Fig 2K).

The above finding merited a more complete investigation. However, the limited amounts of ICSM18 available to us precluded extensive dose-escalation experiments. We therefore performed CA1 injection into *tg*a*20* (females) and into prion protein-ablated mice (*Prnp*°^/^° females) for control. We found that ICSM18 induced lesions in *tg*a*20* but not in *Prnp*°^/^° mice (S3A and S3B Fig, upper row, S3C and S3D Fig). No lesions were observed after injection of the POM2 antibody which had been previously established to be innocuous [15].

We considered that injection into an anatomical area with a denser and distinct neuronal population might influence lesion induction. We therefore administered ICSM18 (6 μg) into the hippocampal CA3 region close to the dentate gyrus of *tg*a*20* mice. Here, we found a more robust induction of neurotoxicity in contrast to the CA1 injection (S3A and S3B Fig, lower row, S3D Fig). Using the CA3 injection coordinates, we then performed injections at 2 μg and 6 μg ICSM18 into BL10 mice, in order to estimate the boundaries of a safe dose. A dose of 6 μg ICSM18 induced significant hyperintense lesions in contrast to 6 μg of BRIC222 (Fig 2D, upper row, Fig 2K; S1 Table), subsequently confirmed by histological analysis (Fig 2E and 2F, upper row). No lesions were found after injection of 2 μg ICSM18 (Fig 2J and 2K). Accordingly, we were able to estimate 3.1 μg as the upper limit of the ICSM18 intracerebrally injected safe dose (S2B Fig).

To test for gender and strain-dependent effects, we injected 6 μg ICSM18 into BL6 males and females. We identified a trend towards more severe lesions in BL6 compared to BL10 females, but the differences were not significant (Fig 2D–2F, middle and lower row, Fig 2K).

As the binding interface of ICSM18 and POM1 overlaps we next asked if the deleterious effect of both antibodies is comparable. Quantitative analysis revealed no significant difference between the toxicity of ICSM18 and POM1, whereas injection of POM1 pre-incubated with mrPrP did not induce a lesion (Fig 2G–2I and 2K).

### Brain penetration of antiprion antibodies after stereotaxic injection

In order to obtain information about the tissue penetration of intracerebrally injected antiprion antibodies, we used a previously described approach for chronically administering antiprion antibodies [10]. We used antibody POM2 [24], which recognizes the PrP^C^ octapeptide repeat motif, conjugated to the fluorescent dye Cy5. POM2 was previously shown to be nontoxic, and elevated interstitial fluid pressure within the lesions may increase tissue penetration [25]. Therefore, POM2 could help evaluate the diffusion of high-affinity antiprion antibodies in the absence of tissue damage. According to previous studies, diffusion is inversely proportional to the density of antigen [26] but independent of affinity as long as the dissociation constant *K*_*d*_ is <10nM.[27].

**Fig 3:**
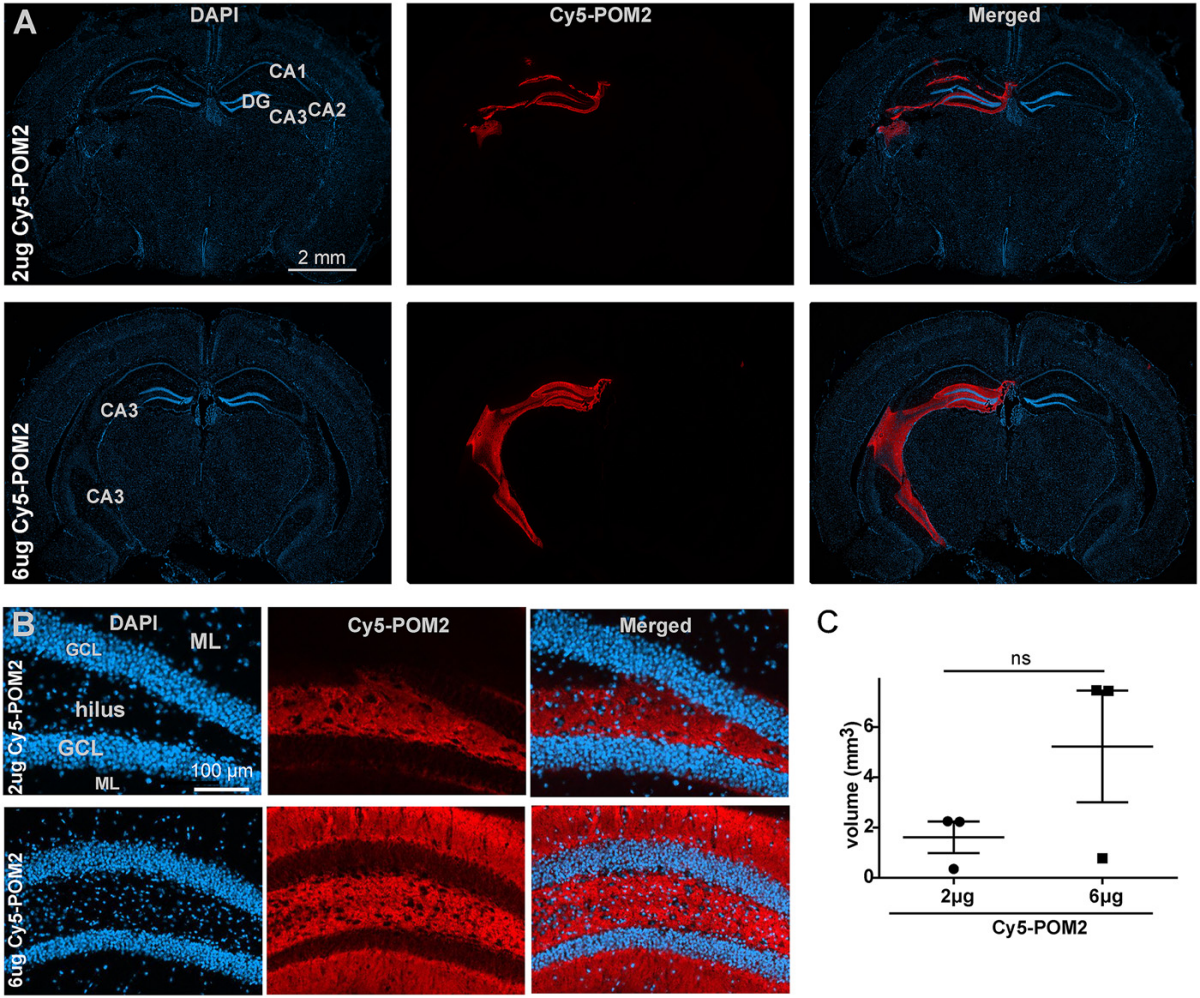
Penetration of monoclonal antibodies after stereotaxic injection. (**A**) Representative frozen sections of mouse brains at 24h p.i. of Cy5-POM2 conjugate (2 μg or 6 μg) into the CA3 region. Conjugated holoantibody diffuses within a well-defined region of the hippocampus, but did not penetrate into other neuroanatomical regions. DG: dentate gyrus; CA: cornu ammonis. (**B**) Higher-magnification images of the dentate gyrus illustrate the distribution of Cy5-POM2 around the neurons of the granular cell layer (GCL), the molecular layer (ML), and the hilus. (**C**) Mean distribution volumes of 1.8 mm^3^ and 5 mm^3^ were found after administration of 2 μg and 6 μg of Cy5-POM2 conjugate, respectively.

As in previous experiments, we administered the Cy5-POM2 conjugate (2 or 6 μg in a volume of 2μl) into the CA3 region. Frozen sections were obtained 24h post injection. We found labeled antibody to be distributed mainly within the hippocampus (Fig 3A and 3B). As reported previously, the fluorescence pattern showed a relatively sharp border between labeled and unlabeled tissue rather than a continuous gradient [26, 28]. This property allowed us to define a distribution volume of 1.8 and 5 mm^3^ for the injection of 2 and 6 μg, respectively (Fig 3C). This observation is in line with the known dependence of antibody diffusion velocity on concentration [29]. The estimated distribution volumes appeared larger than the lesional volumes at the investigated doses (e.g. 1.2 vs. 5 mm^3^, respectively, for antibody D13 at 6 μg). In order to better understand this relationship, we determined the ratio between lesional and distribution volume at 6 μg (S1 Table), and found it to be 25% for D13 and 4–8% for ICSM18. In both dose groups, only minimal Cy5 fluorescence was detected within the brain in one out of three injections (Fig 3C). This phenomenon may possibly result from accidental intravascular injection, and may provide another explanation for the variability in the size of the lesions (e.g. 12 μg of D13).

### Brain destruction after chronic exposure to antiprion antibodies

Because of limited penetration, intrathecal injection of high-affinity antiprion holoantibodies will unlikely represent the optimal therapeutic choice against prion diseases, a disease that affects the entire brain. Antibody derivatives with a more efficient tissue penetration are preferred, including lower-molecular weight binders [29]. Furthermore, stereotaxic intracerebral administration of a single dose of antiprion antibodies does not represent a realistic approximation of clinical drug administration. To better simulate potential clinical situations, we administrated continuously a single-chain variable fragment (scFv) version of antibody POM1 (Kd: 800nM [24]) using an osmotic minipump. The osmotic minipumps were loaded with 75 μg POM1 (0.6 μg/μl) and dispensed antibody at a rate of 0.25 μl/h for 21 days to *tg*a*20*.

**Fig 4:**
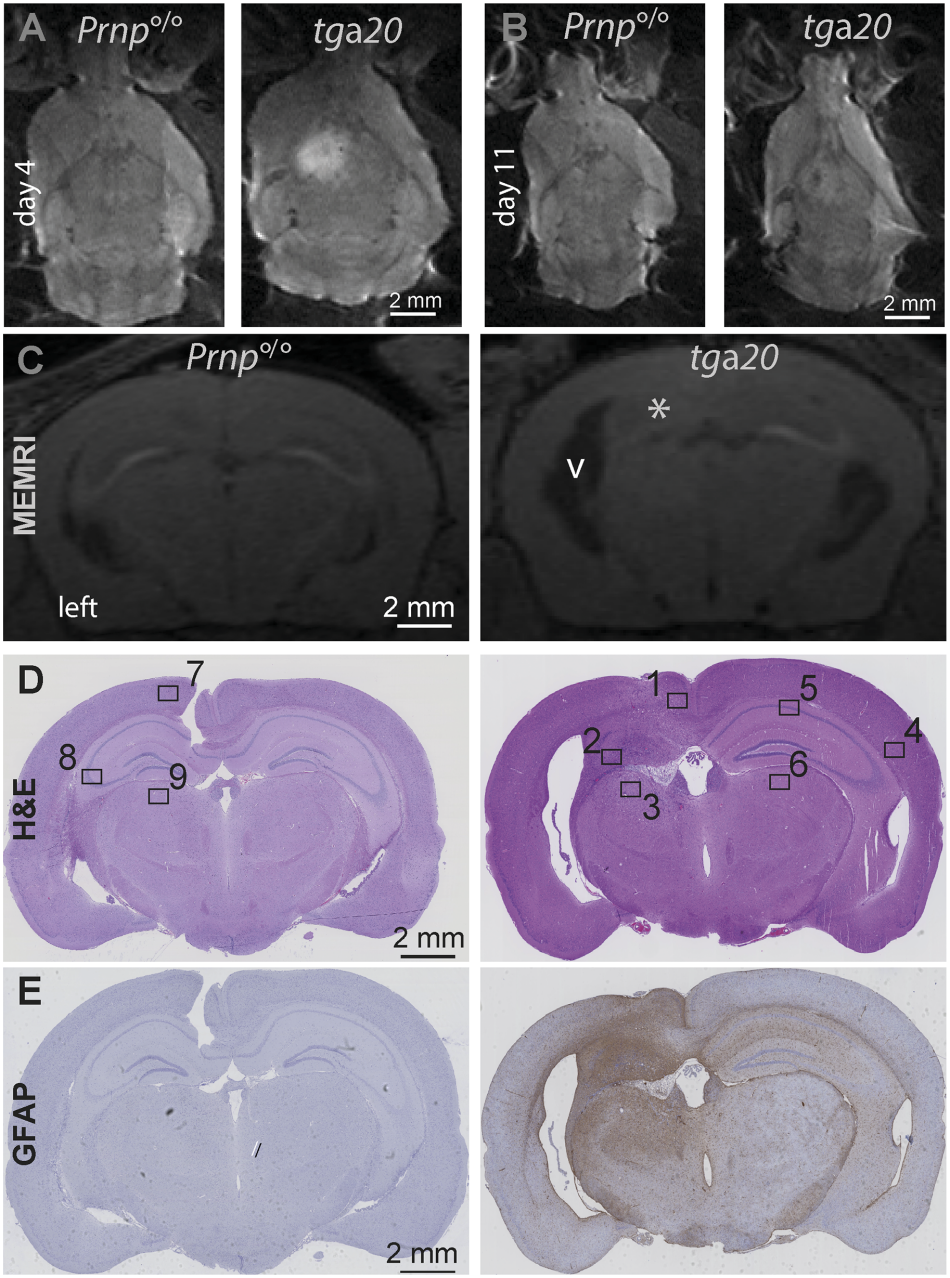
Tissue damage after chronic exposure to the toxic antiprion antibody POM1. (**A**) Representative DWI images of mouse brains 4 days after the implantation of a mini-osmotic Alzet pump delivering a single-chain variable fragment (scFv) of POM1. A large region of restricted diffusion became visible in *tg*a*20* mice (right), whereas no lesion was detected in *Prnp*°^/^° mice (left). (**B**) Images of the same mice as in panel A, at 11 days p.i. The hyperintense signal in the *tg*a*20* mouse was no longer visible, indicating that the acute phase of tissue damage had subsided. (**C**) Coronal manganese-enhanced MRI (MEMRI) images of the *tg*a*20* mice shown in panels A and B. A reduction of the MEMRI signal was visible in the dentate gyrus (asterisk) and CA1 region. Moreover, there was a conspicuous enlargement of the ventricles (v) indicating parenchymal loss. No lesion was detected in *Prnp*°^/^° mice. (**D**) Representative HE sections from the mice shown in panels A at 21 p.i. Gross damage to the brain architecture, especially to the hippocampus of *tg*a*20* mice. Numbered rectangles: regions magnified in supplemental S4 Fig. (**E**) Low-magnification micrographs of glial fibrillary acidic protein (GFAP) stainings of consecutive sections, illustrating tissue damage in both hemispheres, particularly in the hippocampal region.

Echoplanar DWI at 4 days after pump implantation showed that POM1 induced hyperintense lesions spreading from the area around the implanted cannula. No lesions were seen in *Prnp*°^/^° mice subjected to the same procedure (Fig 4A). The hyperintense signal regressed 11 days post implantation (Fig 4B). Manganese enhanced magnetic resonance imaging (MEMRI, Fig 4C) and HE-stained histological sections (Fig 4D and S4 Fig) confirmed the presence of large cystic lesions at 21 days post implantation. Volumetric quantification of the lesions seen by MEMRI image indicated that 9±3% of the total brain volume in *tg*a*20* mice, and 1±0% in *Prnp*°^/^° mice, was affected. This quantification may underestimate the overall tissue damage, as conspicuous astrogliosis and microgliosis were found in both hemispheres indicative of generalized brain involvement (Fig 4E and S4 Fig).

## Discussion

Klöhn *et al*. were unable to reproduce the initial report [14] of D13 toxicity, and concluded from their experiments that "PrP antibodies do not trigger mouse hippocampal neuron apoptosis" [16]. However, the results presented here indicate that this conclusion is not universally correct. We found that both the ICSM18 and D13 antibodies are neurotoxic in paradigms seemingly identical to those used in the studies of Klöhn *et al*. In all likelihood the lesions described here may have been missed by Klöhn *et al*., because their chosen dosage approximated but did not reach the minimal toxic concentration. This outcome vividly depicts the necessity to perform accurate dose-escalation studies when studying the toxicity of potential therapeutics.

Beyond the obvious dosage-related issues, several additional confounders and pitfalls may have contributed to the inability of others to detect toxicity. For example, the purity and age of antibody batches can affect toxicity in ways that may be subtle and difficult to control. Next, we have found that variations in the injection coordinate can influence the volume of lesions. Possible explanations for this finding include the selective vulnerability of different neuronal populations in the hippocampus to antiprion antibodies. Similar differences are seen in other pathological conditions such as ischemia [30], toxins [31] and various neurodegenerative diseases [32]. Another explanation is that extracellular fluid is drained differentially from distinct brain sites. Interestingly, we found a trend towards larger lesion in BL6 mice in contrast to the BL10 mice used by Klöhn *et al* [14], which probably also affected the detection of ICSM18 induced neurotoxicity. In fact, these two mouse wild type strains have been found to display different responses to pathogens in other settings [33, 34].

Whilst we could reproduce the initial report of D13 neurotoxicity, we found the toxicity threshold to be higher than originally reported by Williamson *et al*. This quantitative discrepancy is suggestive of different biological activities of the antibody batches used in previous studies and may be related to the specific methodologies used for protein generation and purification.

The estimated hypothetical upper safe dose was higher for ICSM18 than for D13. A difference in biological activities of both antibodies cannot be fully excluded, as it was not possible to generate both antibodies in our laboratory. Thus, our data may not be sufficient to directly compare the two antibodies. However, the POM1 antibody, whose epitope overlaps with that of ICSM18, shows an equivalent response to the latter. We therefore speculate that there is a relation between the binding epitope and dose-response relationship, which in line with the observation that toxicity is epitope-dependent [15].

Our results are not surprising considering that neurotoxicity was identified as a potential hazard of antiprion immunotherapy by many laboratories [13–15]. Since toxicity was found to be reproduced with single-chain variable fragments and F(ab)_1_ fragments of antibodies, it seems related to "on-target" interaction with PrP, rather than antibody effector functions. Depending on the ratio between the dosage required for effectiveness and the toxicity threshold, such vulnerability may represent a surmountable obstacle for the development of therapeutic antibodies.

The first patients envisaged for clinical studies will likely be those suffering from sporadic CJD (sCJD), since this is the most frequent prion disease in humans. sCJD is hypothesized to begin with the spontaneous misfolding and aggregation of prion protein within the brain. Accordingly, informative pre-clinical studies should prove the efficacy in disease models where prions are inoculated intracerebrally. However, a cumulative dose of 8 mg of ICSM18 (injection of 2 mg intraperitoneal twice weekly) was shown to be ineffective against intracerebral prion inoculation [8]. It is plausible that the antibody passes through the blood brain barrier in sufficient amounts only at the terminal stage of the disease, as was found in the case of antibody 31C6 [9]. Thus, it may be necessary to select an intracerebroventricular route of administration. The only published study on delivering anti-prion antibody via this route (31C6) used 336 μg (0.5 μl/h, 14 days, 2 mg/ml) to achieve a significant but small prolongation of survival in prion inoculated mice [10]. However, when we administered POM1 in a similar dose range intracerebroventricularly using osmotic minipumps, we found massive destruction of brain matter. These results along with the lack of preclinical data on the chronic toxicity of ICSM18 mandate particular caution with respect to the possible intrathecal administration of ICSM18.

We considered the possible impact of species-specific *Prnp* polymorphisms on antibody toxicity. Although we have only investigated the interaction of ICSM18 with murine PrP^C^, similar effects are likely to occur in humans since the atomic pivots contributing to the binding were found to be similar in the two species [20]. The minor differences between the binding of ICSM18 to the human and murine α3 helix are likely due to the inferior resolution of mutational scanning compared to crystallographic epitope mapping.

After chronic scFvPOM1 delivery by osmotic minipumps, we found lesions similar in size and distribution to the previously described diffusion pattern of antiprion antibodies [10, 13]. In contrast, after a single stereotactic injection we identified lesions in only 4–25% of the total antibody distribution volume. Hence chronic administration induces cumulative damage over days [10] with lesion size being superimposable to the distribution volume, even at relatively low antibody concentrations (0.5 μg/μl scFvPOM1). While sobering, these results do not indicate that antiprion immunotherapy is inherently unsafe. In fact, we reported earlier that 5 of 12 “POM” antibodies tested against PrP^C^ did not show any cytotoxicity in organotypic slice cultures, and the innocuousness of the octapeptide repeat ligand POM2 was confirmed *in vivo* with up to 6 μg in PrP^C^-overexpressing *tg*a*20* mice [15]. Moreover, other antibodies including 31C6, 44B1, and 110 [9, 10], did not show untoward effects at high doses in preclinical efficacy studies.

A meta-analysis of many published studies (Table 2) points to a relationship between binding epitopes and neurotoxicity in vivo, with toxicity appears to be strictly dependent on well-defined epitopes of the globular domain of PrP^C^. Antibody 31C6 was reported to bind residues 143–149 on the α1 helix of PrP^C^, thus encompassing three amino acids with close intermolecular contacts to POM1 and ICSM18. Also antibody D18, reported to be innocuous, binds to these residues. Neither of these two antibodies is described to engage epitopes on the α3 helix (e.g. Lys^204^). Hence engagement of the α3 helix may be important in mediating neurotoxicity. However, this interpretation is currently speculative since D18 has not been tested in a rigorous dose-escalation study, nor has high-resolution epitope mapping been applied to these antibodies. Conversely, antibodies binding the flexible tail of PrP^C^ seem to be generally well-tolerated and, in our view, are more amenable to the development of safe and effective antiprion immunotherapies.

**Table 2:** PrPC epitopes and toxicity of anti-PrPC antibodies.

	Epitopes (structures)				Method for epitope mapping	In vivo toxicity	
Antibody	OR	CC2	H1	H3		yes	no
POM2	Four repetitive epitopes within 57–88	-	-	-	Peptide competition ELISA [24]	[15], §	
106	59–66; 83–90	-	-	-	ELISA with deletion mutants, spotted peptide arrays [35]	[10]	
110	59–65; 83–89	-	-	-	ELISA with deletion mutants, spotted peptide arrays [35]	[10]	
4H11	*	*	*	*	*		[13]
P	-	96–105	-	-	Peptide phage display [36]	[16]	[14]
D13	-	96–104	-	-	Peptide phage display [37]	[16]	[14], [15], §
ICSM35	-	96–109	-	-	Peptide ELISA [38]	[16]	
D18	-	-	133–157	-	Peptide phage display [37]	[14], [16]	
31C6	-	-	143–149	-	ELISA with deletion mutants, spotted peptide arrays [35]	[10]	
44B1	**	**	**	**	ELISA with deletion mutants [35]	[10]	
ICSM18	-	-	143–145; 148; 151–152	204	Co-crystallization (Fab) [19]	[16]	§
POM1	-	-	138–147	204/208/212	Co-crystallization, NMR spectroscopy [15]		[15], §

OR: Octapeptide repeats; CC2: Charged cluster; H1: Helix 1; H3: Helix 3

* This antibody was reported to bind to the octapeptide repeat domain. The primary data and epitope mapping method were not disclosed [13].

** Discontinuous epitope, including the region within residues 155–231 [35].

^§^ The present study.

POM1 was toxic to wild-type organotypic cerebellar slices at 167 nM (25 ng/μl) [15] which is ca. 100-fold lower than the toxic concentration in vivo (20 μM; 3 μg/μl). This is unsurprising, since organotypic slices were exposed continuously to POM1 and diffusion from the site of injection would massively reduce the half-life of the antibody at the site of injection, whereas little degradation of the antibody is expected to occur in vitro. For antibody D13, whose epitope is similar to that of POM3, the situation may be more complex. In organotypic slices, D13 showed limited intrinsic toxicity at relatively high concentrations. However, when administered at lower concentration, D13 protected slices from POM1 toxicity. This behavior is compatible with the hypothesis that D13 is a partial competitive agonist which competes with POM1 for a pathogenic target.

In summary, these data illustrate that the efficacy profile (i.e. the curative effectiveness versus the potential toxicity) of antiprion antibodies is complex and depends both on intrinsic factors such as, crucially, the nature of the engaged epitope, and extrinsic factors such as the route of administration. Detailed analyses and mapping of the involved epitopes and—most importantly—appropriate dose-escalation studies in vivo are prerequisite not only for preparing clinical trials in humans, but also to avoid the reporting of contradictory, confusing, and potentially misleading results.

## Materials and Methods

### Mice

Mice were housed under specific pathogen-free conditions. Female and male inbred mouse strains C57BL/6JOlaHsd1 (BL6) were bred in-house. C57BL/10 (BL10) were purchased from Harlan or from the Jackson laboratory. Coisogenic BL6.129-*Prnp* ^o/o^, BL6.129-*Prnp* ^o/o^ *-tg*a*20* ^+/+^ (*tg*a*20*) mice were bred on a mixed 129S2/SvHsd and C57BL/6JOlaHsd1 background [17, 39, 40].

### Ethics statement

Animal care and all experimental protocols were in accordance with the “Swiss Ethical Principles and Guidelines for Experiments on Animals”, and approved by the Animal Experimentation Committee of the Canton of Zurich (permits 130/2008 and 41/2012). Animal care and protocol guidelines were obtained from http://www.blv.admin.ch/themen/tierschutz/index.html?lang=en and strictly adhered to by the experimenters and animal facility at the institution where the experiments were performed.

### Chemicals and the generation of antibody derivatives and recombinant PrP

All compounds were purchased from Sigma/Aldrich unless otherwise stated. POM [24] and D13 monoclonal antibodies [37] were produced in-house using hybridoma technology and purified by affinity chromatography using protein G sepharose and diluted in PBS. Silver-stained SDS-PAGE gels showed that antibodies were essentially pure. Monoclonal ICSM-18 (produced based on hybridoma technology and purified by affinity chromatography) was provided in limited amounts from Simon Hawke and dialyzed against PBS prior to intracerebral injection. Recombinant mouse PrP was generated in E. coli, purified by affinity chromatography and on-column oxidized and refolded into its native state [41]. BRIC222 was purchased from American Research Products (Waltham MA 02452, USA).

### MEMRI and DWI

For MEMRI, mice received five intraperitoneal injections of MnCl_2_ (40 mg/kg, 20 mM in H_2_O and bicine, pH 7.4) at 12h intervals [42]. The final injection was administered immediately after the stereotaxic injection. For imaging acquisition, the mice were placed under isoflurane anesthesia at 4h, 24h and 72h post-surgery. Initially, the mice were placed on a bed equipped with a mouse whole-body radio frequency transmitter coil and a mouse head surface-coil receiver and then into the 4.7 Bruker Pharma scan. Body temperature was maintained with a warming blanket. For MEMRI T-1 weighted brain images were obtained using a 3D gradient-echo sequence with the following parameters: TR: 15 ms, TE: 2.5 ms, flip angle: 20 deg, average: 10, Matrix: 265/265/126 Voxel, Field of View: 2x2.56x2 cm^3^, acquisition time: 1h, Voxel size: 78x100x156 μm^3^. For DWI, routine gradient echo sequences with the following parameters were used: TR: 300 ms TE: 28 ms, flip angle: 90 deg, average: 1, Matrix: 350 x 350, Field of View: 3 x 3 cm, acquisition time: 17 min, voxel size: 87x87 μm^3^, slice thickness: 700 μm^3^, Isodistance: 1400 μm^3^ and b values: 13, 816 s/mm^2^. For chronic treatment, diffusion weighted images were assessed with an echo planar sequence with: TR: 7500, TE: 44.6, Voxel size: 0.1x0.1x0.7 mm, b value: 500 s/mm^2^.

### Quantification of MRI scans

Quantification was performed with ParaVision software (Version 5, Opl3, Bruker). Lesions were quantified by assessing two regions of interest (ROIs), corresponding to the lesion and the total cerebellar (or hippocampal) area. ROIs were set for each optical slice of the data set. In order to quantify hippocampal lesions with MEMRI scans, the volume of non-affected CA3 was measured. ROIs were set on the ipsilateral and contralateral sides of injection. Volumes for each ROI were calculated by multiplying the sum of the ROI area by the voxel height. Data are presented as the lesion volume divided by the total cerebellar volume or hippocampal volume. For hippocampal lesions assessed by MEMRI scans, data are presented as CA3 volume (mm^3^), separated by ipsilateral versus the contralateral side. For the statistical analysis of experiments involving the comparison of three or more data sets, we used the one-way ANOVA with Dunnett’s post-hoc test. The two-tailed unpaired Student’s t-test was used to compare two data sets. The results are displayed as mean ±s.d. *: P<0.05; **: P<0.01; ***: P<0.001, ****: P<0.0001.

### Benchmark dose analysis

Dose response analysis and the benchmark dose relation were calculated with benchmark dose software (BMDS) 2.4 (United States Environmental Protection Agency).

Dose-response relations were fitted to the dataset (log_10_ values) using the following equation:

$$\text{(Y}\left[\text{dose}\right]{\text{ = intercept+v*dose}}^{\text{n}}\text{/}\left({\text{k}}^{\text{n}}{\text{+dose}}^{\text{n}}\right).$$

The intercept was defined as the mean lesion volume after control injection, which equals 0.05 mm^3^. The mean lesion volume was calculated by volumetric quantification of the hyperintense signal after BRIC222 and rmPrP preincubation injections at the different doses.

The v value of the equation was set to the different maximal lesion volumes in order to model the different scenarios. For the BMD analysis of D13 and ICSM18 we used the mean and maximal lesion volume from the 12 μg D13 injection group (parameters from the log_10_ values), 3.68 mm^3^ and 40 mm^3^ respectively, and 453 mm^3^ the mean brain volume of wild type (BL6) mice [43], which is the theoretical maximal possible response. Additionally for the BMD analysis of ICSM18 we used the maximal lesion volume after 6 μg ICSM18 injection (0.4 mm^3^).

The adverse effect level, also referred to as the benchmark response (BMR), equals 0.15 mm^3^, and was established at 0.1 over the mean lesion volume after control injection. Of note, we used the absolute definition of the adverse effect level [22]. The BMR was fitted to the graph with the equation Y[dose] = 0.15. Benchmark doses (BMD) represent the intercept of the BMR line with the fitted curve. To estimate the upper limit of the D13 intracerebrally injected safe dose, the lower 95% confidence limit of the BMD was calculated.

### Stereotaxic injections

Mice were anaesthetized with isoflurane and placed in a motorized stereotaxic frame controlled by software with a three-dimensional brain map, allowing for real-time monitoring of needle placement (Neurostar). The skull was exposed by a midline incision and a small hole was drilled using a surgical drill. The needle (Hamilton, pstAS, gauges 26 s) was then mounted in an electronic micro-injector unit and was placed for cerebellar injection at the following lambda coordinates: AP -2.3 mm, ML 0 mm, DV 2 mm, for CA1 injection at: A/P: -2 mm, ML: 1.3 mm, DV: -1.4 mm from Bregma or for CA3 injection at the following Bregma coordinates: AP– 2 mm, ML ±1.7 mm, DV 2.2 mm, angle in ML/DV plane 15°. Antibodies (2 μl) were injected at a flow rate of 0.5 μl/min. After termination of the injection, the needle was left in place for 3 min. The surgical wounds were sutured, and mice received an injection of buprenorphinum (0.1 mg/g body weight).

### Histology and immunohistochemistry of formalin-fixed, paraffin embedded tissue

Mice were euthanized after the last scan and the brains were fixed in 4% formalin. Cerebella or a 4 mm coronal section from the posterior cortex were paraffin embedded and 2 μm coronal step sections (standard every 100 μm) were cut, deparaffinized and routinely stained with hematoxylin and eosin. For immunohistochemistry, sections were deparaffinized through xylol and graded alcohols. Then, heat-induced epitope retrieval in the microwave was performed in 10 mM citrate buffer at pH 6. Sections were incubated for 1h in blocking buffer (0.2% Triton X-100, 10% normal goat serum dissolved in phosphate-buffered saline: PBS) and incubated with rabbit polyclonal either anti-Caspase3 (5 μg/ml, Milipore), rabbit polyclonal anti-GFAP (10 μg/ml, Dako) or rat monoclonal anti-CD68 (10 μg/ml, Abd Serotec) diluted in blocking buffer at 4°C overnight. For 3,3′-diaminobenzidine tetrahydrochloride hydrate (DAB) immunohistochemistry, sections were washed with PBS and incubated for 1 hour at room temperature with the specific biotinylated secondary antibody (10 μg/ml, Vector Laboratories). Sections were then washed with PBS and incubated with horseradish peroxidase-avidin/biotin complex (Vector Laboratories). For visualization, sections were incubated for 5 minutes in DAB (0.5 mg/ml) dissolved in phosphate buffer (0.1 M, pH 7.4), and DAB conversion into an insoluble brown product was induced with hydrogen peroxide. For immunofluorescence detection, brain sections were incubated with fluorescently-labeled secondary antibody (1 μg/ml Alexa Fluor 488). In the final PBS wash, 4,6-diamidino-2-phenylindole dihydrochloride (DAPI; Molecular Probes) was added, and sections were mounted with Fluor Save (Calbiochem). For analysis, pictures were taken with a FluoView FV10i Confocal Laser Scanning System.

### TUNEL stainings

During apoptotic cell death, DNA is fragmented by endonuclease activity. Free hydroxyl groups at the 3’end can be labeled by the enzyme Terminal deoxynucleotidyl transferase (TdT) with labeled nucleotides, a method known as Terminal Uridine Deoxynucleotidyl Transferase dUTP nick end labeling (TUNEL) staining. The in situ Cell Death Detection kit (Roche) was used according to the manufacturer’s instructions. In brief, two micrometer sections of paraffin-embedded brain tissue were deparaffinized, rehydrated and incubated with proteinase K (PK) 20 μg/ml for 10 min at 37°C to break through the formalin fixation induced protein cross links. Then the sections were incubated with the working-strength terminal deoxynucleotidyl transferase (TdT) enzyme and digoxigenin labeled dUTP for 60 min at 37°C, following staining with fluorescently labeled anti-digoxigenin antibody. Sections were counterstained with DAPI.

### Detection of antibody distribution with Cy5 labeled POM2 and volume quantification

The entire antibody POM2 was labeled with using the Cy5 mAB labeling kit (GE Healthcare Amersham) and injected into the hippocampus of 3-month-old mice. Twenty-four hours post-antibody injection, mice were euthanized and 4 mm coronal sections of the posterior cortex were embedded in Hanks balanced salt solution and frozen with liquid nitrogen. Cryosections (10 μm thick) were prepared and stained with DAPI (Molecular Probes) and mounted with Fluor Save (Calbiochem). Every tenth section was imaged with the virtual microscope AxioScan Z1 (Zeiss). Regions of interest (ROI) defining the area of the antibody were set for every image and volumes were calculated by multiplying the total ROI area by the slice thickness.

### Osmotic minipump implantation

Osmotic minipumps (Alzet Model 2004 0.25 μl/h) were filled with antibody diluted in PBS, according to the manufacturer’s instructions. Filled pumps were placed in PBS at 37°C for 24h. *Tg*a*20* was anaesthetized with isoflurane and placed in a motorized stereotaxic frame as described above. Next, the skull was exposed via an incision along the midline. Using blunt surgical dissection, a paraspinal, subcutaneous pouch was generated, in which the pump was positioned. A MRI compatible polyether etherketone medical microtubing (Alzet) was connected to the pump and positioned at the following Bregma coordinates: AP -0.22 mm, ML 0.9 mm, DV 2.5 mm and fixed to the skull with glue (AdheSe One F Viva Pen Refill and Heraeus Kulzer FLOWline). Mice were housed individually after surgery. Post intervention, the mice were treated with subcutaneous injections buprenorphinum (0.1 mg/kg, Reckitt Benckiser, Switzerland), funixin (5 mg/kg, Provet AG, Switzerland) and glucose 5% (20 μl/kg). Sulfadoxinum (2 ml of 24%, Veterinaria AG, Switzerland) and sugar (30 g) were added per liter of drinking water for one week post-surgery.

## Supporting Information

S1 Table Lesion volumes in dependence of brain region, strain, gender, and antibody dose. (PDF)

S1 Fig Surface stereoview of human PrP(121–230). Yellow and cyan: POM1 and ICSM18 interfaces, respectively (PDB accession codes: 4DGI and 2W9E). Magenta: overlap between the POM1 and ICSM18 interfaces. The interface encompasses the nine residues His140, Phe141, Gly142, Ser143, Asp144, Tyr145, Glu146, Asp147, and Lys204. Blue ribbon: polypeptide backbone. Interfaces are delineated by residues with ≤5 Å distance in the complexes comprising hPrP(121–230) and the respective F(ab) fragments. Structural images were prepared with the program PyMOL (www.pymol.org).
(TIF)

S2 Fig **(A) Hypothetical benchmark dose analysis using log**_**10**_ **values of the lesion volumes at different doses after D13 injection (data as in Fig 1).** Curves represent dose response relations fitted to the dataset (Y[dose] = 0.05 + v*dose^n^ / (k^n^+dose^n^)). To model the different scenarios for the maximal lesion volume, the v value of the equation was set to the different values: purple = 3.68 mm^3^, blue = 40 mm^3^, and green = 453 mm^3^. A hyperintense signal on DWI with a mean volume larger or equal to 0.15 mm^3^ was detemined as the adverse effect level upon administration of toxic antiprion antibodies in contrast to control injections (dashed red line, Y[dose] = 0.15 mm^3^), representing the benchmark response (BMR). The benchmark dose (BMD) is defined as the dose inducing the BMR (intercept point). The vertical lines indicate the BMD values corresponding to the different dose response values (purple: 5.4 μg, blue line: 3.9 μg and green line: 3.7 μg). The upper limit of the safe dose is provided by the lower 95% confidence interval of the BMD (horizontal lines below the graph: purple: 3.3, blue: 1.9 μg and green: 1.5 μg). Lesion volumes depicted on a log_10_ scale. (**B**) Dose-response models based on the log_10_ values of volumetric lesion quantification of ICSM18 injections (data as in Fig 2). Curves of different colors correspond to different assumptions of the maximal lesion volume (v). Black, purple, blue, and green: fitted values of 0.4 mm^3^, 3.63 mm^3^, 40 mm^3^, and 453 mm^3^ were assumed for the maximal lesion volume, respectively. BMD for ICSM18; black: 5.8 μg, purple: 5.9 μg; blue: 5.9 μg; green: 5.9 μg, based on the BMR (dashed red line). The horizontal lines below the graph correspond to the lower 95% confidence interval of the BMD (light-black: 3.1 μg, light-purple: 3.1 μg, light-blue: 3.1 μg, light-green: 3.1 μg). Lesion volumes depicted on a log_10_ scale.
(TIF)

S3 Fig **(A) Representative HE section 48h after stereotaxic injections of 6 μg ICSM18 or BRIC222 into the CA1 region (upper row) or CA3 region (lower row) of *tg*a*20* female mice.** Rectangles: regions magnified in panel B. (**B**) Higher magnification revealing neuronal damage after injection of 6 μg ICSM18 (red rectangle), but not after injection of 6 μg BRIC222 (yellow rectangle). Neuronal damage after injection into the CA3 region was more severe than in the CA1 region. (**C**) No lesions were found after injection of 6 μg ICSM18 into the CA3 region of *Prnp*°^/^° mice (48h p.i.). (**D**) Significant lesions were induced by ICSM18 injection into the CA3 and CA1 region of female *tg*a*20* mice, in contrast to injection into PrP deficient mice and to isotype control injection. Lesions in the CA3 region are more consistent, reflected in a higher significance level. Values are depicted on a log_10_ scale. Multi column comparison (first three samples and last three samples) with one-way Anova with Tukey’s post-hoc test, comparing of two samples with two-tailed Student’s *t*-test, ***P<0.001, **P<0.01, *P<0.05, ns: not significant.
(TIF)

S4 Fig High-magnification HE, GFAP and CD68 stainings of cortical, hippocampal, and thalamic areas from the chronically scFvPOM1-treated *tg*a*20* and *Prnp*°^/^° mice shown in Fig 4. The *tg*a*20* brain shows extensive damage with neuronal cell loss and vacuolation indicative of edema (yellow arrowhead). Some vacuoles were opaque and morphologically reminiscent of the spongiform changes occurring in prion infections (yellow asterisks). GFAP staining illustrates astrogliosis in all three areas and in both hemispheres. The proliferation of microglial cells is evidenced by CD68 immunostaining and most prominent in the thalamic region and cortex around the vacuoles (yellow asterisks). Numbers refer to the rectangles depicted in Fig 4.
(TIF)
