# Supplementary material for: Differential Toxicity of Antibodies to the Prion Protein
Source: PLoS Pathog. 2016 Jan 28;12(1):e1005401. doi: 10.1371/journal.ppat.1005401 (PMC4731068; doi:10.1371/journal.ppat.1005401)
Supplement: S1 Table — (PDF) [file ppat.1005401.s001.pdf]

Supplementary Table 1: Lesion volumes in dependence of brain region, strain, gender, and antibody dose.

**Antibody**	**D13**			**ICSM-18**								**POM1**
Affinity (nM)	3.3 (41)*			0.13 (17)*								0.58 *
Brain region	CA1	CA1	CA1	CA1	CA1	CA3	CA3	CA3	CA3	CA3	CA3	CA3
Dose (µg) / (µM)	2 / 6.6	6 / 20	12 / 40	6 /20	6 /20	2 /6.6	6 /20	6 / 20	6 / 20	6 / 20	6 / 20	6 / 20
Strain **	BL6	BL6	BL6	BL10	*tga20*	BL10	BL10	BL6	BL6	*tga20*	*Prnp*$^{o/o}$	BL6
Prnp gene dosage	+/+	+/+	+/+	+/+	+++	+/+	+/+	+/+	+/+	+++	Null	+/+
Gender	m	m	m	f	f	f	f	m	f	m	f	m
Lesioned vs. injected mice	0/5	4/5	4/5	1/4	3/3	0/3	3/4	3/4	4/4	3/3	0/4	3/4
Lesion volume (mm^3 avg ± SD)	0.03 ± 0.02	3.74 ± 3.36	12.27 ± 17.24	0.09 ± 0.09	1.54 ± 1.64	0.04 ± 0.04	0.26 ± 0.15	0.41 ± 0.17	0.65 ± 0.59	4.40 ± 2.21	0.03 ± 0.02	0.23 ± 0.15
Parameters for log_{10} values (mm^3 avg ± SD)	0.02 ± 2.24	1.23 ± 13.49	3.68 ± 9.12	0.05 ± 3.55	0.95 ± 2.88	0.02 ± 2.69	0.33 ± 5.62	0.4 ± 1.41	0.21 ± 1.82	3.98 ± 1.78	0.03 ± 1.86	0.08 ± 1.82
Mean lesion volume / antibody distribution at 6 µg (%)	-	25	-	-	-	-	7	8	4	-	1	2

* Data from reference (22) reproduced for convenience.

** BL6 and BL10 mouse strains have been found to display different responses to pathogens in other settings (31, 32).

+++: overexpressor.
