# Supplementary figures and images for: Differential Toxicity of Antibodies to the Prion Protein
Source: PLoS Pathog. 2016 Jan 28;12(1):e1005401. doi: 10.1371/journal.ppat.1005401 (PMC4731068; doi:10.1371/journal.ppat.1005401)

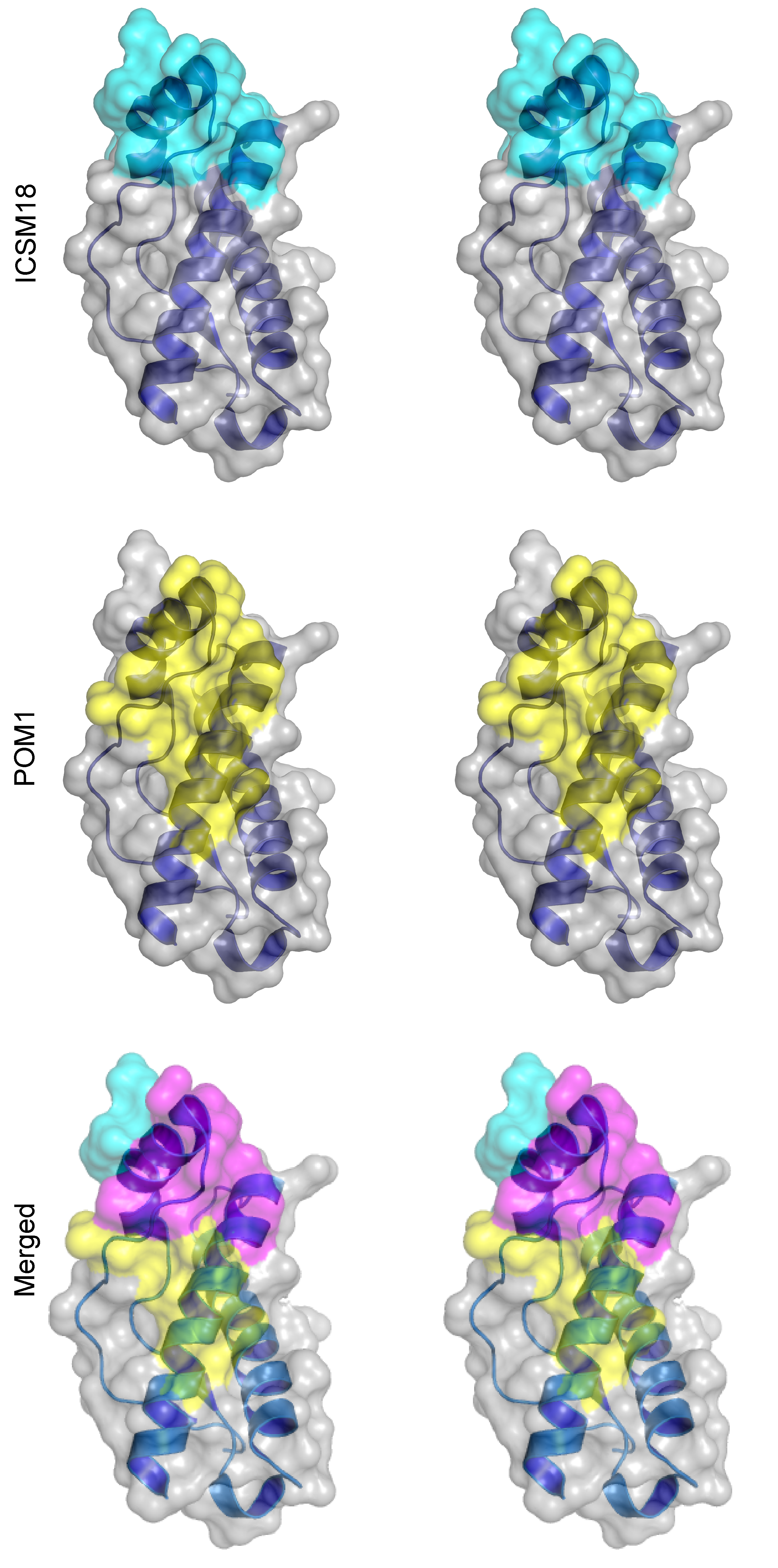

Supplement: S1 Fig — Yellow and cyan: POM1 and ICSM18 interfaces, respectively (PDB accession codes: 4DGI and 2W9E). Magenta: overlap between the POM1 and ICSM18 interfaces. The interface encompasses the nine residues His140, Phe141, Gly142, Ser143, Asp144, Tyr145, Glu146, Asp147, and Lys204. Blue ribbon: polypeptide backbone. Interfaces are delineated by residues with ≤5 Å distance in the complexes comprising hPrP(121–230) and the respective F(ab) fragments. Structural images were prepared with the program PyMOL (www.pymol.org). (TIF) [file ppat.1005401.s002.tif]

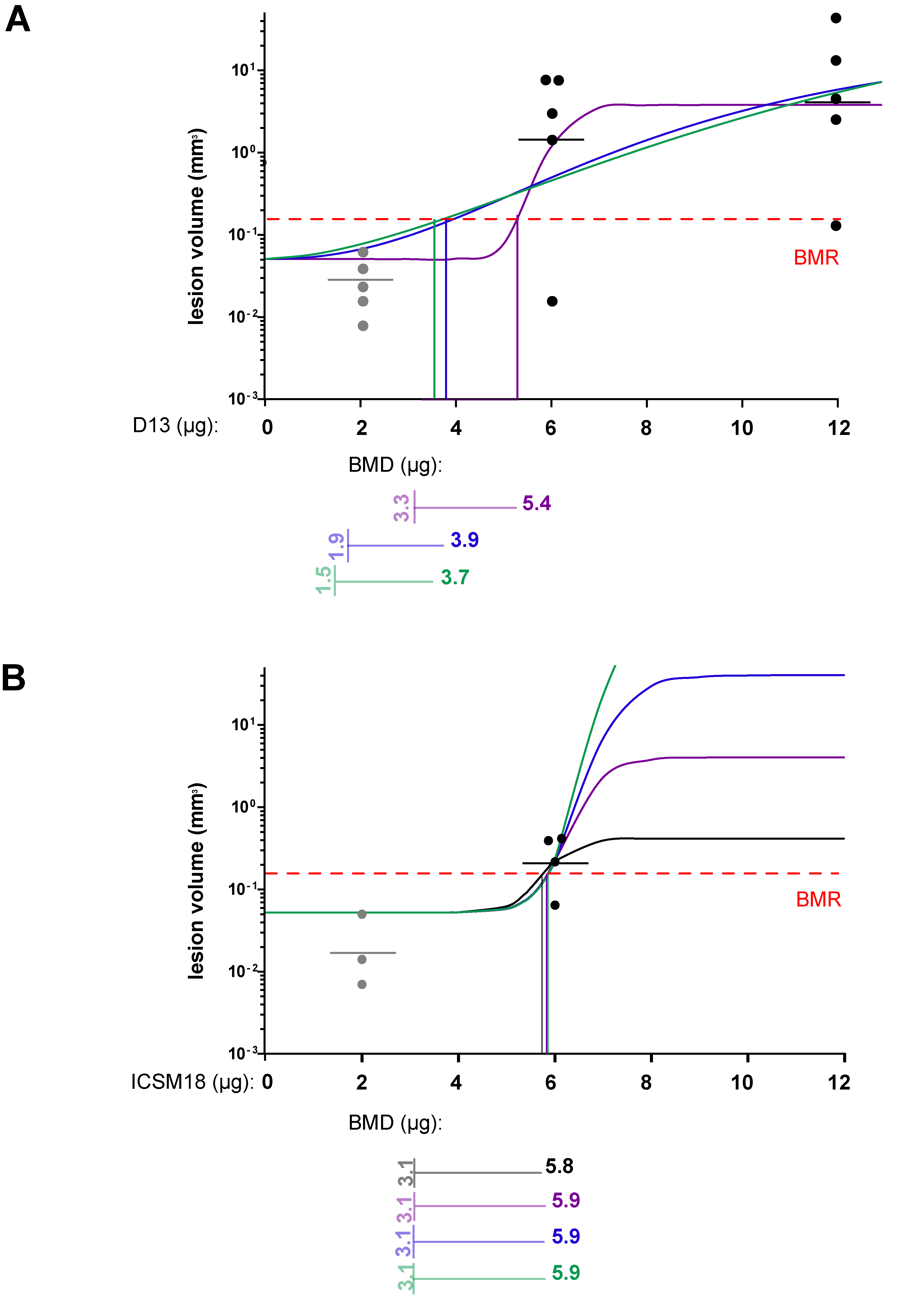

Supplement: S2 Fig — (A) Hypothetical benchmark dose analysis using log 10 values of the lesion volumes at different doses after D13 injection (data as in Fig 1). Curves represent dose response relations fitted to the dataset (Y[dose] = 0.05 + v*dosen / (kn+dosen)). To model the different scenarios for the maximal lesion volume, the v value of the equation was set to the different values: purple = 3.68 mm3, blue = 40 mm3, and green = 453 mm3. A hyperintense signal on DWI with a mean volume larger or equal to 0.15 mm3 was detemined as the adverse effect level upon administration of toxic antiprion antibodies in contrast to control injections (dashed red line, Y[dose] = 0.15 mm3), representing the benchmark response (BMR). The benchmark dose (BMD) is defined as the dose inducing the BMR (intercept point). The vertical lines indicate the BMD values corresponding to the different dose response values (purple: 5.4 μg, blue line: 3.9 μg and green line: 3.7 μg). The upper limit of the safe dose is provided by the lower 95% confidence interval of the BMD (horizontal lines below the graph: purple: 3.3, blue: 1.9 μg and green: 1.5 μg). Lesion volumes depicted on a log10 scale. (B) Dose-response models based on the log10 values of volumetric lesion quantification of ICSM18 injections (data as in Fig 2). Curves of different colors correspond to different assumptions of the maximal lesion volume (v). Black, purple, blue, and green: fitted values of 0.4 mm3, 3.63 mm3, 40 mm3, and 453 mm3 were assumed for the maximal lesion volume, respectively. BMD for ICSM18; black: 5.8 μg, purple: 5.9 μg; blue: 5.9 μg; green: 5.9 μg, based on the BMR (dashed red line). The horizontal lines below the graph correspond to the lower 95% confidence interval of the BMD (light-black: 3.1 μg, light-purple: 3.1 μg, light-blue: 3.1 μg, light-green: 3.1 μg). Lesion volumes depicted on a log10 scale. (TIF) [file ppat.1005401.s003.tif]

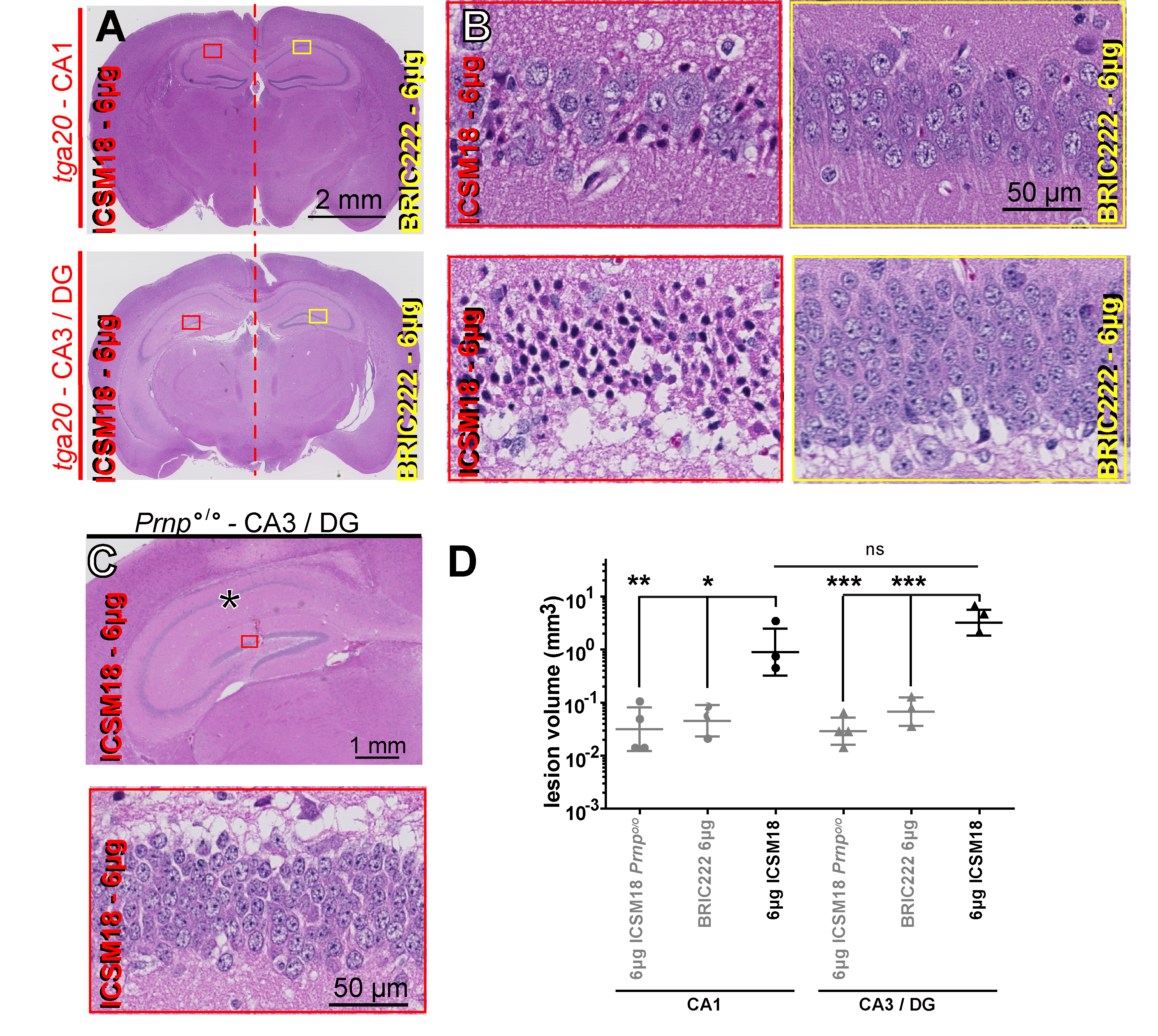

Supplement: S3 Fig — (A) Representative HE section 48h after stereotaxic injections of 6 μg ICSM18 or BRIC222 into the CA1 region (upper row) or CA3 region (lower row) of tga20 female mice. Rectangles: regions magnified in panel B. (B) Higher magnification revealing neuronal damage after injection of 6 μg ICSM18 (red rectangle), but not after injection of 6 μg BRIC222 (yellow rectangle). Neuronal damage after injection into the CA3 region was more severe than in the CA1 region. (C) No lesions were found after injection of 6 μg ICSM18 into the CA3 region of Prnp°/° mice (48h p.i.). (D) Significant lesions were induced by ICSM18 injection into the CA3 and CA1 region of female tga20 mice, in contrast to injection into PrP deficient mice and to isotype control injection. Lesions in the CA3 region are more consistent, reflected in a higher significance level. Values are depicted on a log10 scale. Multi column comparison (first three samples and last three samples) with one-way Anova with Tukey’s post-hoc test, comparing of two samples with two-tailed Student’s t-test, ***P<0.001, **P<0.01, *P<0.05, ns: not significant. (TIF) [file ppat.1005401.s004.tif]

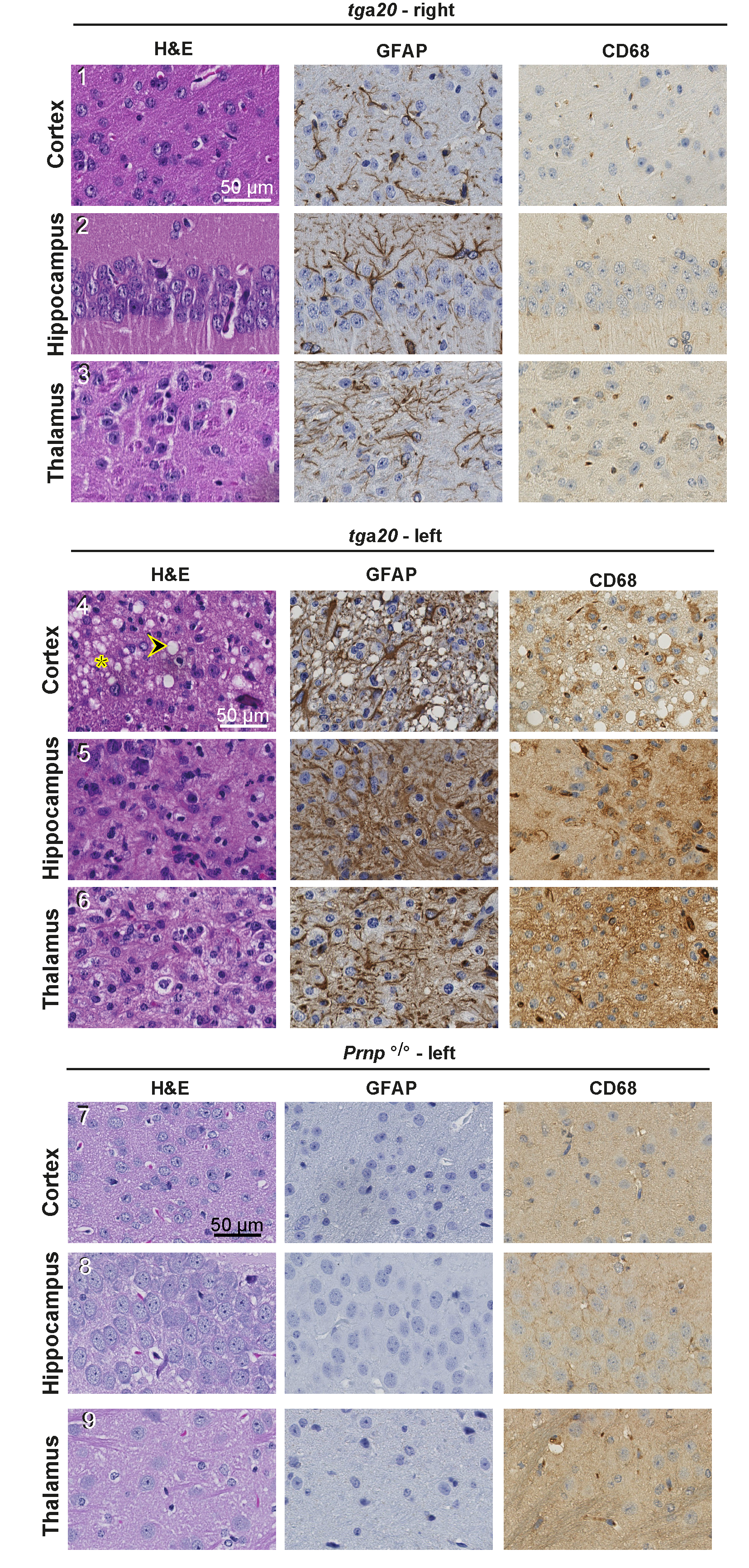

Supplement: S4 Fig — The tga20 brain shows extensive damage with neuronal cell loss and vacuolation indicative of edema (yellow arrowhead). Some vacuoles were opaque and morphologically reminiscent of the spongiform changes occurring in prion infections (yellow asterisks). GFAP staining illustrates astrogliosis in all three areas and in both hemispheres. The proliferation of microglial cells is evidenced by CD68 immunostaining and most prominent in the thalamic region and cortex around the vacuoles (yellow asterisks). Numbers refer to the rectangles depicted in Fig 4. (TIF) [file ppat.1005401.s005.tif]
